# Reconstructing nonlinear dynamic models of gene regulation using stochastic sampling

**DOI:** 10.1186/1471-2105-10-448

**Published:** 2009-12-28

**Authors:** Johanna Mazur, Daniel Ritter, Gerhard Reinelt, Lars Kaderali

**Affiliations:** 1 Viroquant Research Group Modeling, University of Heidelberg, Bioquant BQ26, INF 267, D-69120 Heidelberg, Germany; 2 Discrete Optimization Research Group, University of Heidelberg, INF 368, D-69120 Heidelberg, Germany

## Abstract

**Background:**

The reconstruction of gene regulatory networks from time series gene expression data is one of the most difficult problems in systems biology. This is due to several reasons, among them the combinatorial explosion of possible network topologies, limited information content of the experimental data with high levels of noise, and the complexity of gene regulation at the transcriptional, translational and post-translational levels. At the same time, quantitative, dynamic models, ideally with probability distributions over model topologies and parameters, are highly desirable.

**Results:**

We present a novel approach to infer such models from data, based on nonlinear differential equations, which we embed into a stochastic Bayesian framework. We thus address both the stochasticity of experimental data and the need for quantitative dynamic models. Furthermore, the Bayesian framework allows it to easily integrate prior knowledge into the inference process. Using stochastic sampling from the Bayes' posterior distribution, our approach can infer different likely network topologies and model parameters along with their respective probabilities from given data. We evaluate our approach on simulated data and the challenge #3 data from the DREAM 2 initiative. On the simulated data, we study effects of different levels of noise and dataset sizes. Results on real data show that the dynamics and main regulatory interactions are correctly reconstructed.

**Conclusions:**

Our approach combines dynamic modeling using differential equations with a stochastic learning framework, thus bridging the gap between biophysical modeling and stochastic inference approaches. Results show that the method can reap the advantages of both worlds, and allows the reconstruction of biophysically accurate dynamic models from noisy data. In addition, the stochastic learning framework used permits the computation of probability distributions over models and model parameters, which holds interesting prospects for experimental design purposes.

## Background

Since in 2003 the Human Genome Project released the complete human genome sequence, there is great interest in the complex interplay between different genes and proteins. Instead of focusing on individual cellular components, interest has shifted to the interplay between these components, introducing the view that a biological system is more than the sum of its parts.

One of the most difficult problems in systems biology is the reconstruction of gene regulatory networks from experimental data. This difficulty arises from numerous sources, among them the combinatorial explosion of possible network topologies for a given number of genes, limited information content and high levels of noise in experimental data, limited amounts of data, and the complexity of regulatory processes in cells during transcription, translation and post-translation.

Many approaches have been proposed to infer networks from data, good reviews are, for example, [1-8]. A common method to represent dynamics in biochemical systems are differential equations [9]. Rich mathematical theory has been established for their solution and analysis, and can be exploited [10,11]. Linear differential equation models have been proposed to infer gene regulatory networks [12,13]. These are attractive models due to the low number of parameters and their analytical tractability. However, since biological networks are typically highly nonlinear, linear differential equations are usually not adequate to accurately capture a regulatory network's dynamic behavior [14,15]. Some authors argue that if a system is linearized around a specific point of interest, e.g., a steady state, one may describe the local behavior using linear models [16-20].

To describe more complex dynamic behavior, nonlinear models are needed. Such models can describe nonlinear behavior such as oscillations, multi-stationarity and biochemical switches. Furthermore, by using differential equations which are based on chemical reaction kinetics, model parameters directly correspond to reaction rates, thus models and model parameters can be immediately interpreted biochemically [21].

On the other hand, due to the high-dimensional search space, inference of nonlinear models from data is much more complex than linear system identification, and serious problems with over-fitting and non-identifiability arise.

Nevertheless, nonlinear models are increasingly being used, and are very likely to play an important role in our ability to understand progressively more complex systems in the future. Bongard and Lipson recently published a method that can be used to symbolically infer nonlinear systems without prior specification of a model class, which they applied to simulated data of a three-component model of the *lac *operon [22]. While such a model-free approach is very interesting, it remains to be seen whether the methodology can be extended to larger networks.

Making assumptions about the underlying model class, Spieth *et al. *used S-systems [23,24], generalized linear models [25] and so-called H-Systems and inferred models with up to 10 genes from data, using different search strategies, including evolutionary algorithms [26-28]. A cooperative, coevolutionary algorithm was used by Kimura *et al. *for the inference of S-system models of genetic networks [29]. Perkins *et al. *used partial differential equations to reverse engineer the *Gap *gene network in *Drosophila melanogaster *[30]. Busch *et al. *recently used an approach related to delay differential equations to infer the regulatory network underlying keratinocyte migration [31].

While models based on differential equations provide a quantitative dynamic description of a system under consideration, they completely disregard the stochastic nature of biological data. Linear stochastic differential equations have been proposed for this reason [32], but they still require strong assumptions, and it is unclear if larger, nonlinear stochastic differential equation models of genetic regulatory networks can successfully be inferred from experimental data.

A further difficulty with differential equation models is, that it is not straightforward to compute probability distributions over alternative models or model parameters. This would be most useful in particular if several alternative models fit the data well, and could be used to design additional experiments. Furthermore, such information would make it possible to consider alternative scenarios also in simulation-based perturbation studies, e.g., when interest is on the effect of potential drug candidates. The problems of over-fitting and non-identifiability of models typically encountered with nonlinear differential equation models can be addressed by regularization [20,32,33], or by including additional biological knowledge in the inference process [34,35]. However, the former requires setting a regularization parameter, which is often a nontrivial problem, whereas the latter approach requires a systematic way to include such information in parameter estimation. Both issues can nicely be addressed in a Bayesian framework.

We therefore embed a nonlinear ordinary differential equation (ODE) model, based on chemical reaction kinetics, into a Bayesian framework. Network inference then amounts to evaluating the posterior distribution over models and model parameters, given the experimental data. A related idea has recently been pursued by Steinke *et al. *for linear models [20]. In their paper, the authors use expectation propagation to evaluate the posterior distribution. We combine a *nonlinear *differential equation model based on chemical reaction kinetics with a Bayesian framework, and use a Markov chain Monte Carlo (MCMC) approach to sample from the posterior distribution.

A difficulty with this approach comes from the fact, that it requires solving the differential equations at every step in the Markov chain. To avoid this problem, we adapt the parameter estimation method proposed by Ramsay *et al. *[36] for Markov chains. The authors iteratively fit smoothing splines to the data, and then learn the parameters of the differential equations using a least squares procedure on the slope estimates of the splines and the differential equations. The idea to carry out the optimization on slopes instead of concentrations was first suggested by Varah in 1982 [37]. It was then improved by Poyton *et al.*, who proposed to iterate between spline interpolation and parameter estimation [38]. Ramsay *et al. *further improved this approach by proposing profiled estimation [36]. We adapt their objective function, and sample both model parameters and smoothing factor using a Markov chain. The combination of differential equations with a Bayesian framework proposed in this work allows it to adequately describe the nonlinear dynamic behavior of gene regulatory networks, and to incorporate prior knowledge into the network inference process at the same time. In contrast to simple optimization of the posterior distribution as we pursued in previous work [39], the MCMC approach used here provides confidences on learned parameters and computes probability distributions over alternative network topologies. This can be used to consider alternative future scenarios in simulation, and permits the design of additional, most informative experiments to improve the inference procedure.

## Methods

### Differential Equations Model

We represent gene regulatory networks as directed graphs *G *= (*V*, *E*), with vertices *V *= {*v*_1_,..., *v*_*n*_} corresponding to genes and directed edges *E *corresponding to regulatory interactions. An edge from gene *i *to gene *j *indicates that the product of gene *i*, *x*_*i*_, influences the expression of gene *j *either by activating or by inhibiting it. We assume that different regulators act independently, such that the total effect on the expression of gene *i *can be written as the sum of the individual effects. This clearly is a simplification, and can be generalized by considering products of effects from different genes. Our ODE model is written as(1)

where *x*_*i*_(*t*) is the concentration of gene *i *at time *t*. Furthermore, *s*_*i *_and *γ*_*i *_are basal synthesis and degradation rates for each gene *i*, which in the absence of regulations from other genes determine the dynamic behavior of component *i*. Variable *β*_*ij *_denotes the regulation strength of component *x*_*j *_on *x*_*i*_, and *f*_*ij *_is the corresponding regulation function. *β*_*ij *_> 0 corresponds to an activation, *β*_*ij *_< 0 to an inhibition, and *β*_*ij *_= 0 means that there is no regulation from gene *j *to gene *i*. As regulation functions we use Hill-type functions(2)

where *m *denotes the Hill coefficient and *θ*_*j *_is related to the reaction rate by describing the concentration of *x*_*j *_needed to significantly activate or inhibit expression of *x*_*i*_. To keep the number of parameters small, we use one joint Hill coefficient *m *for all regulations, and use the same threshold parameter *θ*_*j *_for all regulations out of the same gene *x*_*j*_. The regulation functions (2) are obtained from chemical reaction kinetics by considering the binding process of a transcription factor to a promoter a reversible chemical reaction. For a more detailed derivation, see, for example, [40-42].

**Figure 1:**
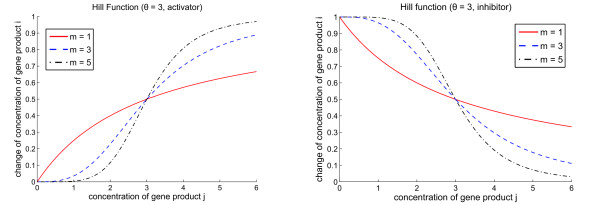
**Regulation functions**. Hill functions *f*_*ij *_for different Hill coefficients *m *= 1, 3, 5. The left plot shows an activation, the right plot an inhibitory effect. The threshold *θ*_*j *_was chosen equal to 3 for both plots, at this concentration of the regulating gene *j*, half the maximum effect on gene *i *is achieved.

Figure 1 shows Hill functions for different Hill coefficients. The left plot shows the case where gene product *x*_*j *_*activates x*_*i*_, the right plot describes an *inhibitory *effect. We note that the formulation used here for inhibitions avoids problems with concentrations possibly becoming negative, as may happen when using the upper function from equation (2) also with *β*_*ij *_< 0, as was done in [39].

### Parameter Estimation of ODE systems

The estimation of model parameters from experimental time series data for differential equation models is typically carried out iteratively in two steps: (1) numerically solve the differential equations for the time interval of interest, and (2) compute an error between experimental data points and model prediction. Initial values and model parameters are then modified to minimize this error. The disadvantage of this procedure is, that the differential equations have to be solved numerically in every iteration of the optimization, which is very time consuming.

As an alternative, Varah proposed a two stage method [37]. In step one, interpolating cubic splines *z*(*t, D*) are fitted to the data *D*. Thereafter, the squared difference between the differential equations and the slope estimates from the interpolation is minimized according to

Here, (*t*_*τ*_, *D*) is the slope estimate from the cubic splines *z*(*t*, *D*), *ω *are the differential equation model parameters, *T *is the number of different time points *t*_*τ *_and *n *is the number of time series to be fitted, for example, the number of different genes in the network.

An obvious drawback of Varah's approach is, that the quality of the parameter estimates can only be as good as the spline fit, which is particularly difficult in case of noisy data. To address this problem, Poyton *et al. *developed a recursive method, called *iterative principal differential analysis*, where the two steps of Varah's method are iterated, and the model predictions are fed back into the spline estimation [38]. Ramsay *et al. *improved this method further using a generalization of profiled estimation to learn the parameters of interest [36].

We adapt this iterative method by simultaneously estimating model parameters *ω *:= (*s*, *γ*, *β*, *m*, *θ*) and smoothing factor *λ *of the smoothing splines. This could be done by minimization of the function(3)

where *d*_*iτ *_denotes the measured data, *T*_1 _is the number of time points in the experimental data and *T*_2 _denotes the number of points to be used in the squared error parameter fitting on the slopes. To adequately describe the dynamics of a system using derivatives, a large number of slope estimates (over time) is required, we will therefore usually have *T*_2 _>> *T*_1_. We note that equation (1) requires concentrations to compute the derivatives, these are taken from the spline interpolation.

### Bayesian Learning Framework

We now address two further problems in parameter estimation, regarding the entire topology of the regulatory network, and variability in experimental measurements. The network topology (which genes have regulatory interactions between them) can either be determined in a separate step prior to parameter estimation, or can be solved implicitly by assuming a fully connected network, and pruning edges with very small regulation strengths afterwards. Determination of the network topology in a separate step has the disadvantage, that edges not included in this prior step cannot be re-introduced in parameter estimation. We therefore use the latter approach, with appropriate regularization to prune many edges during the inference process.

To account for noise in the experimental data, we embed the differential equations into a Bayesian framework. For this purpose, we assume that the measured data *d*_*iτ *_is corrupted by independent mean zero Gaussian noise with variance . The assumption of normally distributed noise is clearly a simplification, which is made here to keep the model simple. Other noise models could be used. We furthermore assume the differences between slope estimates and differential equation predictions to follow a normal distribution with mean zero and variance . We note that the ratio between  and  corresponds to a parameter that weighs the two terms in (3) relative to one another.

The assumption of Gaussian noise leads to the likelihood(4)

which is equivalent to equation (3) up to log-transformation and scaling.

Since we are interested in the probability distribution over the model parameters *ω*, the smoothing factor and the variances  and , given the experimental data *D*, we use Bayes' theorem to obtain(5)

where  is given by the likelihood (4),  is a prior distribution on the model parameters, and *p*(*D*) is a normalizing factor which is independent of *ω*, *λ * and . For simplicity, we treat the variance parameters  and  as user parameters, which are set in advance and not sampled.

### Inclusion of Prior Knowledge

The prior distribution *p*(*ω*, *λ*) on the model parameters can be used to include available biological knowledge on the system under consideration into the network inference process, as demonstrated, for example, by Wehrli and Husmeier [35]. It is a huge advantage of the Bayesian framework that it allows the easy and systematic integration of such expert knowledge. If no such detailed knowledge is available, one can resort to very general assumptions, such as sparsity of the interaction network [43] or rough estimates of reasonable ranges for parameters.

We assume independent prior distributions for the different model parameters, and suggest to use gamma priors

for the synthesis and degradation rates *s*_*i *_and *γ*_*i*_, for the Hill coefficient *m *and the threshold parameters *θ*_*j*_. The parameters *a *and *r *are scale and rate parameters of the gamma distribution, respectively, and Γ denotes the Gamma function

This choice of prior ensures that the parameters are positive, and will not become too large. Since the smoothing factor *λ *ranges between zero and one, we use a beta prior

on *λ*. The parameters *a' *and *b' *are positive shape parameters of the beta distribution, and B is the Beta function

To reflect the assumption of sparsity of gene regulatory networks, we use a prior based on the L_q _norm [44] for the interaction strengths *β*_*ij*_, *i*, *j *= 1,..., *n*:(6)

for *β *∈ ℝ and *q*, *s *> 0, where (*q*, *s*) is the normalizing factor

**Figure 2:**
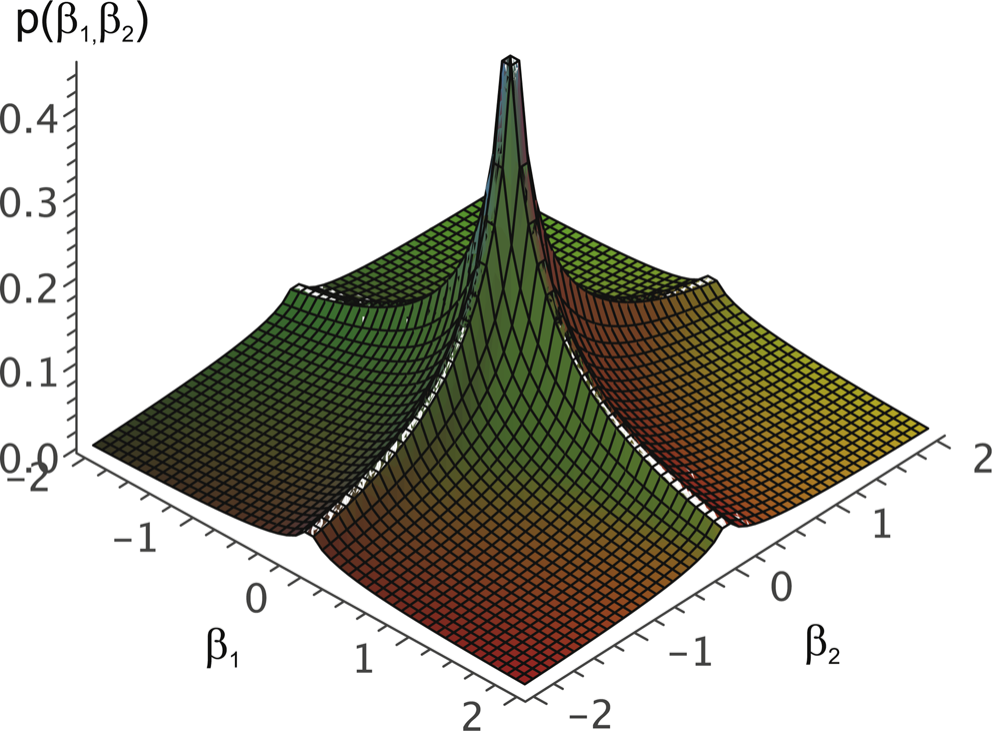
**L_q _Prior**. Plot of the two-dimensional L_q _prior *p*(*β*_1_, *β*_2_):= L_q_(*β*_1_; *q*, *s*)·L_*q*_(*β*_2_; *q, s*) for *q *= 0.5 and *s *= 1. It can clearly be seen how this prior favors points (*β*_1_, *β*_2_) where one of the two components is approximately zero over points at the same distance from the origin with both *β*_1_, *β*_2 _≠ 0.

For *q *= 2, equation (6) is a normal distribution, for *q *= 1 it corresponds to the Laplace distribution. Values of *q <*1 enforce stronger sparsity constraints, as can be seen in Figure 2 for the two-dimensional case with *q *= 0.5 and *s *= 1. In comparison with the prior proposed in [45] and used in network inference in [39], we avoid the numerical integration of the prior required in these publications, and obtain similar sparseness constraints.

### MCMC Sampling from the Posterior

The posterior distribution (5) can now be maximized using, for example, gradient based methods, simulated annealing or genetic algorithms. However, since multiple parameter combinations, corresponding to alternative network topologies, may explain the data equally well, we sample from *p*(*ω*, *λ*|*D*) using Markov chain Monte Carlo. This way, full distributions over each parameter are available, and can be used, for example, to consider different likely topologies, and to design experiments that will resolve ambiguities. This would not be possible with simple maximization approaches.

To sample from *p*(*ω*, *λ*|*D*) we use an iterative approach. First, we sample the model parameters *ω *using the Hybrid Monte Carlo algorithm (HMC), with fixed smoothing factor *λ*. HMC has originally been proposed by Duane *et al. *for problems arising in quantum chromodynamics [46], and has been introduced to the general Bayesian statistical community by Neal [47]. The method samples points from a given *n*-dimensional distribution *p*(*η*) by introducing momentum variables *ρ *= (*ρ*_1_, *ρ *_2_,..., *ρ *_*n*_) with associated energy *K*(*ρ*), and iterative sampling for the momentum variables from *K*(*ρ*) and following the Hamiltonian dynamics of the system *H*(*η*, *ρ*):= -ln *p*(*η*) + *K*(*ρ*) in time. Doing so, HMC generates a sequence of points distributed according to *p*(*η*), and can avoid the random walk behavior typically observed with the Metropolis Hastings algorithm [47].

As the second step, we sample the smoothing factor *λ *using Metropolis Hastings [48,49], with *ω *fixed to the values sampled in the previous step. Pseudocode for our iterative sampling procedure is given in Table 1.

**Table 1:** Iterative Markov chain Monte Carlo Algorithm

**Algorithm 1 **Iterative Hybrid Monte Carlo and Metropolis Hastings algorithm
**Require: **desired distribution *p*(·), starting value (*ω*^0^, *λ *^0^), proposal distribution *q*_*λ *_(·|*λ *^(*t*)^), number of leapfrog steps for HMC *L*, proposal distribution for stepsize ϵ of leapfrog steps *q*_ϵ_(·), standard deviation *σ*_*ρ *_for the sampling of the momentum variables *ρ*, number of Markov chain samples *T*
1: *t *← 0
2: **while ***t *<*T ***do**
3: Sample from *q*_ϵ_(·)
4: Sample from for all *i *∈ {1,..., *n*}
5: Perform *L *leapfrog steps with stepsize starting at state (*ω*^(*t*)^, *ρ *^(*t*)^)
6: Store resulting candidate state in
7: Sample *u*_1 _from (0, 1)
8: *α*_1 _← min {1, exp *H*(*ω*^(*t*)^, *ρ*^(*t*)^) - *H*)}
9: **if ***u*_1 _<*α*_1 _**then**
10: *ω*^(*t*+1) ^←
11: **else**
12: *ω*^(*t*+1) ^← *ω*^(*t*)^
13: **end if**
14: Sample from *q*_*λ*_(·|*λ*^(*t*)^)
15: Sample *u*_2 _from (0, 1)
16:
17: **if ***u*_2 _<*α*_2 _**then**
18: *λ*^(*t*+1) ^←
19: **else**
20: *λ*^(*t*+1) ^← *λ*^(*t*)^
21: **end if**
22: Append (*ω*^(*t*+1)^, *λ*^(*t*+1)^) to Markov chain
23: *t *← *t *+ 1
24: **end while**
25: **return **Markov chain

### Evaluation of Reconstructed Networks

To evaluate reconstructed networks, we summarize the Markov chains by using the mean value of the chain for each model parameter, after excluding points from the burn-in phase of the chain. This is of course a very crude simplification, which we take to allow for an automated, quantitative evaluation of reconstructed networks. Obviously, in case of, for example, bimodal distributions, the mean will be located somewhere between the two modes, possibly in a region with very low probability mass. We therefore emphasize here that the full set of points sampled can and should be analyzed in more detail.

To quantitatively evaluate inferred networks, we use receiver operator characteristic (ROC) and precision to recall (PR) analysis, and summarize these using the area under the curve (AUC). For two-class classification problems (e.g. edge present/not present), ROC curves plot sensitivity against 1-specificity for varying thresholds on the predictor (for example, absolute value of average edge weight *β*_*ij*_), whereas PR curves similarly plot precision against recall (= sensitivity). The AUC is then simply the area under the ROC or PR curve, and on a scale from 0 to 1 provides a single number to measure the quality of a predictor. We note that changing the threshold in ROC and PR curves corresponds to different thresholds for edge pruning in reconstructed networks.

In our case, we want to distinguish between three classes - no edge, activation, or inhibition. Therefore, we map the three-class problem onto a two-class problem as indicated in Table 2. With this approach we calculate sensitivity, specificity and precision, to calculate the area under the ROC and PR curves (AUC_ROC _and AUC_PR_). We point out that for our three-class problem, for a random network, the average expected AUC value will not be 0.5 as in the two-class case, but will vary between zero and 0.39 for AUC_ROC _and between zero and 0.5 for AUC_PR_, depending on the number of non-existing edges in the reference network. For a mathematical proof we refer to Additional file 1.

**Table 2:** Classification Matrix for ROC/PR Evaluation

		**predicted**		
		**positive link**	**negative link**	**non-existent link**
	
	positive link	TP	FP	FN
	
**actual**	negative link	FP	TP	FN
	
	non-existent link	FP	FP	TN

Mapping of three-class classification problem (no edge present, positive regulation, negative regulation) onto two-class ROC/PR evaluation. TP: True Positive, TN: True Negative, FP: False Positive, FN: False Negative.

### Implementation

We implemented our algorithm in Matlab, Release 2008b (The Mathworks), using the spline and statistics toolboxes. Computations were carried out on a Linux cluster with dual-processor 3.1 GHz XEON quadcore machines with 32 GB RAM, running each Markov chain in a single thread (no additional parallelization). The Matlab code is available on request from the authors.

## Results

### Simulated Five Gene Network

**Figure 3:**
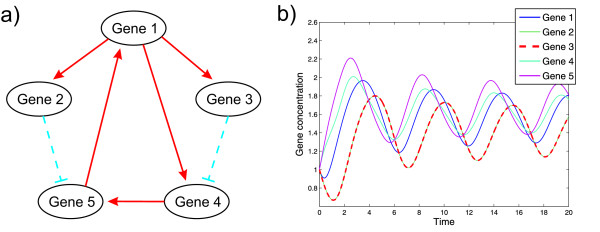
**Gold Standard Topology and Simulated Data**. (a) True network of the DREAM 2 challenge #3 five gene time series data, showing the bio-engineered interactions between the five genes artificially inserted into yeast. (b) Time course of simulation with model in arbitrary time and concentration units, for the simulated five gene model. Different numbers of equidistant time points from this data were used for network reconstruction in the simulation study. The time courses of gene 2 and gene 3 are almost the same.

We first evaluated our approach on a simulated five-gene network. This allows it to systematically study the performance of network inference under varying levels of noise and dataset sizes, while the true network topology is known. We simulated data using the network topology shown in Figure 3a. Since this is the topology also underlying the experimental data used later, this allows a direct comparison of simulation results with inference results on real experimental data.

Data was simulated using the differential equation model (1), with synthesis and degradation rates *s *= (0.2, 0.2, 0.2, 0.2, 0.2) and *γ *= (0.9, 0.9, 0.9, 1.5, 1.5) for the five genes. The threshold parameter of the Hill functions was set to *θ *= (1.5, 1.5, 1.5, 1.5, 1.5), with Hill coefficient *m *= 5. The parameters for the regulation strength were set to *β*_*ij *_= 2 for activations, *β*_*ij *_= -2 for inhibitions, and zero if there was no interaction between to genes, compare Figure 3a.

Data was simulated by numerical integration of the differential equations in Matlab using the function ode45. Simulated data shows oscillations for all genes, see Figure 3b. To simulate the typical setting in network inference, where only a limited number of noisy measurements are available, we evaluated our network reconstruction approach using different numbers of time points subsampled equidistantly from the simulated data, and added mean-zero normally distributed noise with different standard deviations to the concentration values. We then used our method to reconstruct the original network from this data. For this purpose, we sampled 110, 000 points for (*ω*, *λ*) using the algorithm described in Table 1, with a burn-in of 10, 000 points. Parameters of the prior distributions were set to *a *= 1, *r *= 2 for the gamma prior on synthesis and degradation rates, *a *= 1.5, *r *= 5.0 for the gamma prior on the Hill coefficient *m*, *a' *= 100, *b' *= 10 for the beta prior on *λ*, and *q *= 0.5, *s *= 1 for the L_q _prior on the regulation strengths *β*_*ij*_. Shape and scale parameters for the gamma priors on the *θ*_*j *_for each gene *j *where chosen such that mean and variance of the priors correspond to mean and variance of the training data. The number of slope estimates *T*_2 _is set to 1000 and the corresponding variance  is set to 1. Furthermore, the variance  is set to *T*_1_/*T*_2_, where *T*_1 _denotes the number of time points.

#### Results on 40 time points

We first describe results for an ideal case with 40 time points and no noise. In that case, mean values for inferred synthesis and degradation rates were *s *= (0.23, 0.20, 0.29, 0.26, 0.15) and *γ *= (1.17, 1.14, 1.33, 1.00, 0.99). Mean value for the Hill coefficient *m *was 4.76, means for the thresholds *θ*_*j *_ranged from 1.38 to 1.78 and the mean smoothing factor *λ *was 0.92. Inferred regulation strengths (mean and standard deviations) are given in Table 3. The large standard deviations for some regulation strengths, e.g., the self-regulation on gene 3 or the regulation from gene 4 to gene 1, indicate that there either are different network topologies which describe the data well, or that the dynamics of the system is insensitive to changes of this parameter.

**Table 3:** Inferred Interaction Strength Parameters for Simulated Data for Dataset with 40 Time Points

To ↓/From →	Gene 1	Gene 2	Gene 3	Gene 4	Gene 5
**Gene 1**	0.53 ± 0.72	-0.22 ± 0.41	-0.10 ± 0.43	1.37 ± 1.16	**0.88 **± **0.74**
**Gene 2**	**1.54 **± **0.78**	0.28 ± 0.42	0.49 ± 0.62	0.33 ± 0.55	0.28 ± 0.53
**Gene 3**	**1.35 **± **0.80**	0.34 ± 0.57	1.07 ± 1.59	0.55 ± 0.63	0.26 ± 0.46
**Gene 4**	**0.23 **± **0.57**	-0.35 ± 0.61	-**0.51 **± **0.64**	0.40 ± 0.69	0.84 ± 0.74
**Gene 5**	-0.01 ± 0.31	-**0.62 **± **0.67**	-0.91 ± 0.69	**0.55 **± **0.81**	0.65 ± 0.63

Learned regulation strength parameters *β *for the simulated dataset with 40 time points. Given are mean and standard deviation of the sampled interaction parameters. True edges which are present in the reference network are indicated in bold.

Precision to recall and receiver operator characteristic analysis of results yield AUC values of AUC_PR _= 0.516 (guessing: 0.14) for precision to recall curves and AUC_ROC _= 0.706 (guessing: 0.358) for sensitivity vs. 1-specificity curves.

To close the circle from the original concentration data over the reconstructed model back to dynamic simulation, we used the mean inferred model parameters to simulate gene concentrations. This simulation shows an accurate match of simulated and experimental data (not shown). It is well known that fitting models to oscillating data, and even more so, reconstructing networks from such data, are extremely hard problems, since models tend to learn a steady state [50]. In spite of this, oscillations were captured with high precision by our approach (see Figure 3b).

#### Effect of Noise and Dataset Size

We next studied the effect of different dataset sizes (number of time points) and different levels of noise in the data on the quality of network reconstruction. For this purpose, we added mean zero Gaussian noise with standard deviations 0.05, 0.1, 0.15, 0.2 and 0.3 to the simulated concentration data, and furthermore subsampled equidistantly from the time series to generate data sets with *T*_1 _= 10, 20, 30, 40, 50, 70, 90, 110, 140, 170 and 200 different time points for each of the five noise levels. Then the network reconstruction was performed as described in the methods section.

**Figure 4:**
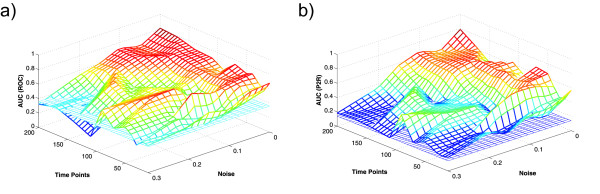
**AUC Values for Simulated Data**. AUC values for different noise levels and different numbers of time points used for network reconstruction. The standard deviation of the noise was varied from *σ *= 0 to *σ *= 0.3, the number of time points from *T *= 10 to *T *= 200. The plots show (a) AUC values under the ROC curve and (b) AUC values for PR curves for varying *T *and *σ*. The blue surface indicates the AUC_ROC _and AUC_PR _values that would follow for random guessing.

Figure 4 shows AUC values for ROC analysis (left) and precision to recall analysis (right) for the different noise rates and number of time points. All runs without noise produced results with very high AUC values. As expected, performance decreases with increasing noise levels and decreasing number of time points. We point out that for oscillations with an amplitude of 0.5 to 0.6, as present in the simulated data, noise with standard deviation 0.3 is already very high and considerably disturbs the oscillations.

### Evaluation on Experimental Data: The DREAM 2, Challenge 3 Dataset

To assess the performance of different reverse engineering approaches, Stolovitzky *et al. *fathered the DREAM (Dialogue on Reverse Engineering Assessment and Methods) initiative [51]. For this purpose, Cantone *et al. *provided in-vivo data on a small, bio-engineered five-gene network, which was posted as challenge #3 within DREAM 2 [52]. This data was generated by inserting new promoter/gene combinations directly into the chromosomal DNA of budding yeast. Two time series of gene expression of the five inserted genes after stimulation were measured using quantitative PCR, measuring 15 time points in 3 minute intervals in time series 1, and 11 time points in 5 minute intervals in time series 2.

Measurements in both time series consist of negative (base 2) log-ratios of the genes of interest to housekeeping genes, we therefore transformed the measured data to recover the original ratios. We used the first time series (3 minute interval data) for network inference, i.e., *T*_1 _= 15.

Figure 3a shows the original engineered network. The topology is the same that we used in the simulation study. We attempted to reconstruct this network from the experimental data alone. For this purpose we ran a Markov chain with 60, 000 steps and a burn-in of 10, 000. The parameters used for the gamma prior for the synthesis rate, degradation rate and Hill coefficient, *T*_2_,  and  were set as described for the simulated data. We set the parameters for the beta prior on the smoothing factor to *a' *= 5 and *b' *= 100.

The hyperparameters for the L_q _prior on the regulation strengths were set to *q *= 1 and *s *= 2. Parameters for the gamma priors on the threshold parameters were manually set to concentrate probability mass near the mean concentration value for each gene individually.

An evaluation of results using the mean of the Markov chain for each parameter, as done for the simulated data, results in AUC values that are equivalent to guessing (data not shown). This might indicate that either the level of noise present in the experimental data is too high, or that the posterior distribution has multiple modes, with the mean being an inappropriate summary statistic. We therefore searched the points sampled for the maximum a-posteriori mode, and evaluate this mode further in the following. Clearly, data from the additional modes are available and can be studied similarly.

**Figure 5:**
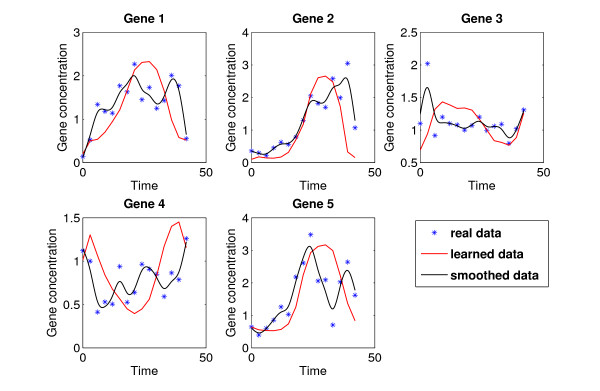
**Learned Dynamics of DREAM 2 Challenge Data**. Plot of the experimental data from the DREAM 2 challenge, in comparison to time courses simulated with reconstructed model parameters. Shown in black is the smoothed data.

The regulation strength parameters for the maximum a-posteriori mode are shown in Table 4. The dynamic behavior and fit of the model prediction to the experimental data is depicted in Figure 5. Obviously, the general dynamics of the original data is well represented, with a moderate amount of smoothing. AUC values of the reconstructed network are 0.532 (guessing: 0.358) for sensitivity vs. 1-specificity, and 0.255 (guessing: 0.14) for precision to recall.

**Table 4:** Inferred Interaction Strength Parameters for DREAM 2 Challenge #3 Data

To ↓/From →	Gene 1	Gene 2	Gene 3	Gene 4	Gene 5
**Gene 1**	1.03	0.03	-0.05	-0.62	**0.12**
**Gene 2**	**1.09**	1.55	-0.09	0.00	-0.10
**Gene 3**	**0.10**	-0.04	0.56	0.16	-0.11
**Gene 4**	-**0.15**	-0.03	-**0.43**	0.26	0.09
**Gene 5**	0.77	-**0.16**	0.01	**0.01**	0.44

Reconstructed maximum-a-posteriori regulation strength parameters *β *for the DREAM 2 challenge #3 data. True edges present in the reference topology are marked in bold.

To assess the quality of this result, we next compared performance of our approach to the performance of other approaches submitted to the DREAM 2 challenge. For this purpose, we computed performance measures as were used in the DREAM 2 challenge for our inferred network, and show results in Table 5. AUC values for this comparison were calculated as described in [51]. Since our method gives us a topology with positive and negative regulation strengths, we have transformed our results to be suitable for the evaluation method used in DREAM 2:

• By skipping the sign and dividing by the largest learned regulation strength for the DIRECTED-UNSIGNED challenge.

• For the two DIRECTED-SIGNED challenges we only took the regulation strengths with the appropriate sign and divide them by the highest absolute regulation strength.

**Table 5:** Results of DREAM 2 Challenge #3 Data Compared to Other Approaches

Challenge	Best submitted	Our method	No self-regulations
DIRECTED-SIGNED-EXCITATORY	AUC = 0.79	AUC = 0.61	AUC = 0.79
	AUC_PR _= 0.72	AUC_PR _= 0.25	AUC_PR _= 0.54

DIRECTED-SIGNED-INHIBITORY	AUC = 0.63	AUC = 0.96	AUC = 0.96
	AUC_PR _= 0.14	AUC_PR _= 0.45	AUC_PR _= 0.45

DIRECTED-UNSIGNED	AUC = 0.73	AUC = 0.56	AUC = 0.79
	AUC_PR _= 0.55	AUC_PR _= 0.30	AUC_PR _= 0.57

Results of the DREAM 2 challenge #3 data of our method (second column) compared to submitted best results from [53] (first column). The third column gives the AUC values for our method when self-regulations are omitted. Our method clearly outperforms all submitted methods in the DIRECTED-SIGNED-INHIBITORY challenge; furthermore, when neglecting self-regulations, we also beat the best submitted method in the DIRECTED-UNSIGNED challenge.

Our method outperforms all submitted results in the challenge DIRECTED-SIGNED-INHIBITORY. One difficulty we observed was, that our approach learned many strong self-regulations of genes, possibly because of an improper balancing of the priors on synthesis/degradation rates and regulation strengths. Since there are no self-regulations in the DREAM 2 challenge #3 data, we provide an additional evaluation when disregarding self-regulations, results are shown in the third column of Table 5. In this case, we not only outperform all submitted approaches in the DIRECTED-SIGNED-INHIBITORY challenge, but also beat the best models in the DIRECTED-UNSIGNED challenge.

## Discussion and Conclusions

We have developed a novel methodological approach to reverse engineer gene regulatory networks from gene expression time series data, and evaluated this approach on both simulated and real gene expression data. The combination of ordinary differential equations and the Bayes' regularized inference technique can be used for the quantitative analysis of complex cellular processes. Non-linear differential equations are able to describe complex dynamic behavior, and a rich mathematical theory for analyzing them is well established. Our method combines these advantages of differential equations with the advantages of a Bayesian framework, which is able to capture noise in data, makes it possible to include biological knowledge into the learning process, and allows the computation of probability distributions over model topologies and model parameters.

The latter is one of the main advantages of the MCMC approach. The information about distributions can be used to make predictions of future states of the network together with confidence intervals on the predictions made. This may allow it to take alternative future scenarios into account, and could be used to design additional most informative experiments that will help to distinguish between corresponding topologies or parameter sets. We therefore think that our approach will be highly useful to elucidate regulatory networks in an iterative procedure with several rounds of experiment, network inference, and experiment design.

In contrast to the usual approach of minimizing an error between experimentally measured concentrations and model predictions, our likelihood function uses the difference between model slopes and experimental slopes. We furthermore integrate a smoothing spline approximation into the likelihood, automatically performing an optimized tradeoff between an accurate representation of the experimental data, and smoothing out noise. Fitting of model parameters on slopes has the advantage that no numerical integration of the model is required in each step of the optimization or sampling. Instead, we must estimate smoothing splines and slopes. This can be carried out much faster than numerical integration, enabling us to use a Markov chain sampler on the posterior distribution instead of plain maximization. We have evaluated our approach on simulated and on real experimental data from a synthetic gene regulatory network. On the simulated data, we have shown that our approach can reconstruct the underlying topology with high accuracy. As expected, performance deteriorates with increasing levels of noise and with decreasing number of different time points available. We emphasize that the simulated example chosen is a difficult learning task due to the oscillations in the data. It is obvious that sufficient data points are required to sample the full dynamics of the oscillating network, and that oscillations quickly break down in the presence of noise.

On the DREAM 2 data, our method yields superior results when compared to other approaches that were submitted to DREAM 2 in the DIRECTED-SIGNED-INHIBITORY and DIRECTED-UNSIGNED categories. Importantly, our analysis shows that there are multiple posterior modes that describe the data well, which may explain a surprising result of the original DREAM 2 challenge: As reported by Stolovitzky et al., none of the submitted models were able to accurately reconstruct the original network topology from the synthetic data [51]. Our results indicate that this might be due to a dense population of local optima, in which network reconstruction approaches looking for a single optimal topology might get trapped and return suboptimal solutions. An obvious conclusion is that further experiments are required to resolve ambiguities in network reconstruction. This emphasizes the need for robust and efficient methods for optimum experimental design. Our sampling approach may be a good starting point for such experiment design, since it analyzes the full distribution over model parameters, and therefore yields information on alternative network topologies and confidence intervals on parameters, which are instrumental to design experiments that elucidate the network topology further.

## Authors' contributions

LK designed the research and methodology. JM and DR wrote the software, analyzed the data, and drafted the paper. GR and LK advised on algorithm design and data analysis, and wrote the final version of the paper. All authors read and approved the final version of the paper.

## Supplementary Material

Additional file 1 **Supplementary material**. Mathematical proof. Click here for file
