# Supplementary material for: Reconstructing nonlinear dynamic models of gene regulation using stochastic sampling
Source: BMC Bioinformatics. 2009 Dec 28;10:448. doi: 10.1186/1471-2105-10-448 (PMC2811124; doi:10.1186/1471-2105-10-448)
Supplement: Additional file 1 — Supplementary material. Mathematical proof. [file 1471-2105-10-448-S1.PDF]

Reconstructing nonlinear dynamic models of gene regulation using stochastic sampling: Supplementary Material: Our ROC analysis

Johanna Mazur, Daniel Ritter, Gerhard Reinelt and Lars Kaderali

In classical two class receiver operator characteristic (ROC) analysis the area under curve (AUC) value gives a measure how well the two classes are separated. In our case this approach is not suitable, since we want to distinguish three classes and evaluate, how well they are predicted. We now describe the confusion matrix underlying our analysis as well as our method for the generation of ROC points and the calculation of the AUC value. After recalling what the AUC value is for guessing in the two class ROC analysis, we calculate the AUC value for guessing in our three class ROC analysis for sensitivity vs. $1-$specificity and precision vs. recall. For more information concerning receiver operator characteristics, see [1].

1 Confusion matrix

In Table 1 the confusion matrix of our three class problem is denoted. An edge is denoted as true positive (TP), if it is a positive or negative link and predicted also as a positive or negative link, respectively. False positives (FP) are all predicted postive or negative links which are not correctly predicted, i.e., either they are non-existent or they have another sign in the reference network. As true negatives (TN) we denote correctly predicted non-existent edges and as false negatives (FN) falsely predicted non-existent edges are defined, i.e., an edge is predicted to be non-existent but it is a positive or a negative link in the reference network.

		**predicted**		
		positive link	negative link	non-existent link
	positive link	TP	FP	FN
**actual**	negative link	FP	TP	FN
	non-existent link	FP	FP	TN

Table 1: Mapping of three-class classification problem (no edge present, positive regulation, negative regulation) onto two-class ROC / PR evaluation.

2 ROC point generation and AUC Calculation

Let B be the set of the predicted interaction parameters, i.e., the edge weights. Further denote by $\beta_{\max}$ the maximum of the absolute values of B. Now we choose a precision factor p and calculate the stepsize $s = \beta_{\max}/p$. Then, for a varying threshold from zero to $\beta_{\max}$ by adding the stepsize s we obtain p thresholds. For each of these thresholds we set the predicted interaction parameters to zero for values inside the interval [- threshold, threshold]. Then we calculate sensitivity, specificity and precision according to the confusion matrix denoted in Table 1 for each of the p thresholds. Then the AUC value is calculated as the integral under the curve described by these points.

3 AUC value for guessing for two class problem

For the two class problem the AUC value for guessing for sensitivity vs. $1-$specificity is known to be 0.5. Assume that one has randomly guessed the edges of a network. If you now have a classifier which gives you sensitivity of 0.8, i.e. 80 percent of true existing edges are found with our classifier, then you also expect to have $1-$specificity of 0.8, i.e. 80 percent of the predicted existing edges are not present in the true network.

The AUC value for guessing for precision vs. recall is also known to be 0.5, since precision is 0.5 (except the case where no existing edge is found, i.e. $\mathrm{TP} = 0$) for varying recall between zero and 1.

4 AUC value for guessing for three class problem

First, we introduce as notations $p(+_L)$ for the probability of a positive learned edge, $p(-_L)$ for the probability of a negative learned edge and $p(0_L)$ for the probability of a non-existent learned edge. Furthermore, we denote by $\mathrm{ratio}(+)$, $\mathrm{ratio}(-)$ and $\mathrm{ratio}(0)$ the ratio between the positive, negative resp. zero edges in the true network. For example, if in the true network there are one third positive edges, one third negative edges and one third zero edges, then $\mathrm{ratio}(+) = \mathrm{ratio}(-) = \mathrm{ratio}(0) = 1$. If two third of the edges are zero edges and one third of the edges are positive, then $\mathrm{ratio}(0) = 2$, $\mathrm{ratio}(+) = 1$ and $\mathrm{ratio}(-) = 0$. Note, that we always have

$$\mathrm{ratio}(0) + \mathrm{ratio}(+) + \mathrm{ratio}(-) = 3 \tag{4.1}$$

in our three class problem.

Remark 4.1. With the method for the ROC point generation described in Section 2 we have two properties of the above defined probabilities:

1. Since we take a kind of symmetric threshold variation, we want to have for randomly generated edge weights
$$p(+_L) = p(-_L). \tag{4.2}$$
2. The aim is now to give a function which depends on $p(+_L)$, $p(-_L)$, $p(0_L)$ and $\mathrm{ratio}(0)$, $\mathrm{ratio}(+)$, $\mathrm{ratio}(-)$, which gives us the points in the ROC graph by varying $p(0_L)$ from zero to one or analogously with (4.2) by varying $p(+_L)$ or $p(-_L)$ from zero to one half.

Furthermore, denote by $p(\mathrm{TP}\,|{+_L})$ the conditional probability for a TP under the condition, that an edge was learned as positive. Similarly, for all other combinations of TP, FP, TN, FN and $+_L$, $-_L$, 0_L. Now assume that one generates randomly with the uniform distribution weights on the

edges. Thereby we assume, that we have infinite many edges to ensure that $p(+_L) = p(-_L)$ and therefore with Table 1 and our assumptions we have

$$\begin{aligned}
p(\mathrm{TP}\,|{+_L}) &= \frac{\mathrm{ratio}(+)}{3} \cdot p(+_L) \\
p(\mathrm{TP}\,|{-_L}) &= \frac{\mathrm{ratio}(-)}{3} \cdot p(+_L) \\
p(\mathrm{FP}\,|{+_L}) &= \frac{\mathrm{ratio}(-) + \mathrm{ratio}(0)}{3} \cdot p(+_L) \\
p(\mathrm{FP}\,|{-_L}) &= \frac{\mathrm{ratio}(+) + \mathrm{ratio}(0)}{3} \cdot p(+_L) \\
p(\mathrm{FN}\,|0_L) &= \frac{\mathrm{ratio}(+) + \mathrm{ratio}(-)}{3} \cdot p(0_L) \\
p(\mathrm{TN}\,|0_L) &= \frac{\mathrm{ratio}(0)}{3} \cdot p(0_L).
\end{aligned} \tag{4.3}$$

Furthermore, we have

$$\begin{aligned}
0 &= p(\mathrm{TP}\,|0_L) = p(\mathrm{FP}\,|0_L) \\
&= p(\mathrm{TN}\,|{+_L}) = p(\mathrm{FN}\,|{+_L}) \\
&= p(\mathrm{TN}\,|{-_L}) = p(\mathrm{FN}\,|{-_L})
\end{aligned} \tag{4.4}$$

and with the classification in Table 1 and together with (4.4) we obtain

$$\begin{aligned}
p(\mathrm{TP}) &= p(\mathrm{TP}\,|{+_L}) + p(\mathrm{TP}\,|{-_L}) \\
p(\mathrm{FP}) &= p(\mathrm{FP}\,|{+_L}) + p(\mathrm{FP}\,|{-_L}) \\
p(\mathrm{TN}) &= p(\mathrm{TN}\,|0_L) \\
p(\mathrm{FN}) &= p(\mathrm{FN}\,|0_L).
\end{aligned} \tag{4.5}$$

With (4.2) we have

$$\begin{aligned}
1 &= p(+_L) + p(-_L) + p(0_L) \\
&= 2p(+_L) + p(0_L).
\end{aligned} \tag{4.6}$$

To calculate sensitivity, specificity and precision we need to know the probabilities for true and false positives as well as for true and false negatives. With (4.3), (4.5) and (4.6) we get

$$\begin{aligned}
p(\mathrm{TP}) &= \frac{\mathrm{ratio}(+) + \mathrm{ratio}(-)}{3} \cdot p(+_L) \\
p(\mathrm{FP}) &= \frac{2\,\mathrm{ratio}(0) + \mathrm{ratio}(+) + \mathrm{ratio}(-)}{3} \cdot p(+_L) \\
p(\mathrm{FN}) &= \frac{\mathrm{ratio}(+) + \mathrm{ratio}(-)}{3} \cdot p(0_L) = \frac{\mathrm{ratio}(+) + \mathrm{ratio}(-)}{3} \cdot \big(1 - 2p(+_L)\big) \\
p(\mathrm{TN}) &= \frac{\mathrm{ratio}(0)}{3} \cdot p(0_L) = \frac{\mathrm{ratio}(0)}{3} \cdot \big(1 - 2p(+_L)\big).
\end{aligned} \tag{4.7}$$

For sensitivity we now obtain

$$\text{sensitivity} = \frac{p(+_L)}{1 - p(+_L)},$$

analogously, for $1-$specificity with (4.1)

$$1 - \text{specificity} \;=\; \frac{\big(\text{ratio}(0)+3\big)\cdot p(+_L)}{\big(3-\text{ratio}(0)\big)\cdot p(+_L)+\text{ratio}(0)}$$

and for precision

$$\text{precision} \;=\; \frac{\text{ratio}(+)+\text{ratio}(-)}{6}. \tag{4.8}$$

We now obtain directly that the AUC value for guessing for the precision vs. recall curve equals the right hand side of (4.8), since it does not depend on $p(+_L)$.

Remark 4.2.
1. The AUC value for guessing for precision vs. recall curve varies between zero and one half. It is zero, if the true network only contains zero edges, i.e., $\text{ratio}(0) = 3$, and it is one half, if the true network does not contain any zero edges, i.e., $\text{ratio}(0) = 0$.
2. The AUC value for guessing for precision vs. recall only depends on the ratio for the zero edges because of (4.1) and *not* on the proportion of the ratios for the negative and positive edges.

To obtain the AUC value for guessing for the sensitivity vs. $1-$specificity curve we have to calculate the area under the curve whose graph G can be written as

$$\begin{aligned} G &= \left\{ \Big(\frac{\big(\text{ratio}(0)+3\big)\cdot a}{\big(3-\text{ratio}(0)\big)\cdot a+\text{ratio}(0)}, \frac{a}{1-a} \Big) \;;\; a \in \Big[0, \frac{1}{2}\Big] \right\} \\ &= \left\{ \Big(x, \frac{\text{ratio}(0)\cdot x}{\text{ratio}(0)+3-3x} \Big) \;;\; x \in [0,1] \right\}. \end{aligned}$$

With integration by parts we now obtain

$$\begin{aligned} \int_0^1 \frac{\text{ratio}(0)\cdot x}{\text{ratio}(0)+3-3x} dx = \frac{\text{ratio}(0)}{\text{ratio}(0)+3} \Big(&-\frac{1}{9} \ln\Big(\frac{\text{ratio}(0)}{\text{ratio}(0)+3}\Big) \cdot \text{ratio}(0)^2 \\ &- \frac{2}{3} \ln\Big(\frac{\text{ratio}(0)}{\text{ratio}(0)+3}\Big) \cdot \text{ratio}(0) \\ &- \ln\Big(\frac{\text{ratio}(0)}{\text{ratio}(0)+3}\Big) - \frac{\text{ratio}(0)}{3} - 1 \Big). \end{aligned}$$

Remark 4.3.
1. The AUC value for guessing for sensitivity vs. $1-$specificity curve varies between zero and approximately 0.39. It is zero, if the true network does not contain any zero edges, i.e., $\text{ratio}(0) = 0$, and it is $2\ln 2 - 1 \approx 0.39$, if the true network only contains zero edges, i.e., $\text{ratio}(0) = 3$.
2. As in the case for guessing for precision vs. recall, the AUC value for guessing for sensitivity vs. $1-$specificity only depends on the ratio for the zero edges and *not* on the proportion of the ratios for the negative and positive edges.

Remark 4.4. By using our notation we will now consider the classical two-class problem. By $p(+_L)$ we now denote the probability of a learned positive and with $p(0_L)$ we denote the probability of a learned negative. Furthermore, we denote by $\text{ratio}(+)$ the ratio for the positives in the true network and with $\text{ratio}(0)$ the ratio for the negatives in the true network. Note that we here have

$$\text{ratio}(+) + \text{ratio}(0) = 2.$$

Then we have for random generated edges

$$p(\mathrm{TP}) = \frac{\mathrm{ratio}(+)}{2} \cdot p(+_L),$$
$$p(\mathrm{FP}) = \frac{\mathrm{ratio}(0)}{2} \cdot p(+_L),$$
$$p(\mathrm{TN}) = \frac{\mathrm{ratio}(0)}{2} \cdot p(0_L),$$
$$p(\mathrm{FN}) = \frac{\mathrm{ratio}(+)}{2} \cdot p(0_L).$$

As in Section 3 we obtain sensitivity $= p(+_L)$, $1-$specificity $= p(+_L)$ and precision $= 0.5$. Thus, we also get the AUC values of 0.5 for precision vs. recall and sensitivity vs. $1-$specificity, respectively. It is important to note here, that, different from our three class ROC analysis, the AUC values do not depend neither on $\mathrm{ratio}(+)$ nor on $\mathrm{ratio}(0)$.

References

[1] Tom Fawcett. An introduction to roc analysis. *Pattern Recognition Letters*, 27:861–874, 2006.
